# Association of Alopecia Areata With Attention-Deficit/Hyperactivity Disorder Stimulant Medication: A Case-Control Study

**DOI:** 10.31486/toj.20.0025

**Published:** 2021

**Authors:** Tyson A. Meaux, Pamela M. McMahon, Glenn N. Jones, Amelia E. Bush, Jaren J. Kennedy, George William Poche

**Affiliations:** ^1^ Department of Dermatology, Louisiana State University Health Sciences Center, New Orleans, LA; ^2^ Pediatric Residency Program, Our Lady of the Lake Children's Hospital, Baton Rouge, LA; ^3^ Department of Family Medicine, Louisiana State University Health Sciences Center, New Orleans, LA

**Keywords:** *Alopecia*, *alopecia areata*, *alopecia universalis*, *amphetamine*, *attention deficit disorder with hyperactivity*

## Abstract

**Background:** Both psychiatric disorders and diverse medications used to treat them have been associated with alopecia. The objective of our study was to investigate the existence of an association between attention-deficit/hyperactivity disorder (ADHD) stimulant medication (ASM) and various types of alopecia.

**Methods:** We conducted a retrospective case-control medical record review of patients between the ages of 6 and 18 years seen in dermatology clinics during a 10-year period. Cases included patients diagnosed with alopecia areata (AA), alopecia totalis (AT), or alopecia universalis (AU). We matched 3 controls on age and sex to each case. We reviewed patients’ medical records for the following medications: lisdexamfetamine, amphetamine/dextroamphetamine, dexmethylphenidate, and methylphenidate. We examined the association between medications used to treat ADHD and diagnoses of AA, AT, and/or AU by calculating a series of odds ratios and 95% CIs.

**Results:** We identified 124 cases (110 with AA, 11 with AT, and 3 with AU) and 372 controls. We found a strong association between AU and ASM use (*P*<0.0071). No relationship between ASM use and other types of hair loss was found.

**Conclusion:** Although the sample size of cases with AU was small, we found a significant association between AU and ASM. While further study is needed, practitioners may consider close monitoring of patients with AA who use ASM for the development of worsening disease and discontinue the medication if the patient experiences an increase in hair loss that appears to be progressing to AU.

## INTRODUCTION

Alopecia areata (AA) is a common, inflammatory, nonscarring type of hair loss that typically presents as well-demarcated patches and can progress to include all scalp hair (alopecia totalis [AT]) or all body hair (alopecia universalis [AU]). Nail findings, such as nail pitting, may also result and are more common in patients with severe disease. AA affects approximately 0.1% to 0.2% of the general population, with both sexes generally thought to be affected equally.^1^ Patients with AA commonly experience psychological distress and impaired quality of life. A 2019 retrospective review of more than 5,500 patients in the United States found that AA was associated with several mental health disorders, and the strongest association was found with attention-deficit/hyperactivity disorder (ADHD).^2^ A Taiwanese study also demonstrated an association between AA and several psychiatric conditions; however, that study found no association between AA and ADHD.^3^ While the existence of an association between psychiatric conditions and AA is not clear, a high stress level and the associated increase in cortisol have been hypothesized to stimulate proinflammatory cytokines, causing an alteration in the immune system and leading to immune attack on hair follicles.^3^

In addition to psychiatric disorders, psychiatric medications, including selective serotonin reuptake inhibitors, antipsychotics, and tricyclic antidepressants, have also been reported in association with AA.^4-6^ One class of psychiatric medication reported in the literature since the 1960s to be associated with hair loss is ADHD stimulant medications (ASM)^7^ such as amphetamine, lisdexamfetamine, and methylphenidate. Hair loss associated with ASM has been reported either as diffuse and rapid, such as in telogen effluvium, or as an exacerbating factor of trichotillomania.^7,8^ The textbook *Stimulant Drugs and ADHD: Basic and Clinical Neuroscience* comments that “the alopecia on stimulants is reversible and temporary, having to do with too many follicles going into the dormant phase in synchrony,”^9^ such as with telogen effluvium. While telogen effluvium may be the culprit in some cases of hair loss associated with ASM, the hair loss time course is difficult to explain when reviewing published case reports. While telogen effluvium hair loss typically occurs 3 months following a trigger,^10^ cases in the literature include diffuse and rapid hair loss 5 days after starting a new ASM, as well as rapid and diffuse hair loss after having taken amphetamines for several years.^11,12^ As in telogen effluvium, some types of AA can present initially with diffuse and rapid shedding of hair. AA incognita and acute and diffuse total alopecia are 2 types of AA that present with diffuse shedding. These 2 types of AA, however, often do not have preexisting localized patches of hair loss.

Given the possible association between ADHD and AA, the uncertain pathomechanism underlying the alopecia associated with ASM, and the various psychiatric medications associated with AA, our study objective was to investigate whether an association exists between ASM use and various types of AA.

## METHODS

This case-control retrospective medical record review was approved by the Louisiana State University Health Sciences Center–New Orleans Institutional Review Board and the Franciscan Missionaries of Our Lady University Institutional Review Board.

We conducted a medical record review of cases (ie, patients with AA, AT, or AU) and controls (ie, patients without AA, AT, or AU) between 6 and 18 years of age seen in dermatology clinics associated with one large health care system between March 15, 2008 and March 15, 2018.

To identify cases, a report of all patients meeting age and visit date criteria with the following diagnoses was generated (*International Classification of Diseases, Ninth Revision* and *Tenth Revision*, respectively): AA (704.01; L63.9, L63.2, L63.8); AT (L63.0); or AU (L63.1). Using a random number generator, 3 control subjects were matched to each case on age and sex from a list of patients without AA, AT, or AU seen in the same dermatology clinics during the same time frame. Patients were excluded if they had ever taken tumor necrosis factor (TNF)-alpha inhibitors (etanercept, adalimumab, infliximab, certolizumab), golimumab, ribavirin, haloperidol, or pegylated interferon, as these medications are reported to cause AA. One control was excluded because the patient was taking adalimumab. No cases were excluded. To ascertain information on ASM exposure, medical records of identified cases and controls were reviewed for the following medications: lisdexamfetamine, amphetamine/dextroam-phetamine, dexmethylphenidate, and methylphenidate.

We calculated a series of odds ratios (ORs) and 95% CIs using SPSS statistical software, version 25 (IBM Corp) to examine the association between ASM and diagnoses of AA, AT, and AU. We analyzed the relationship between sex and ASM with chi-square analysis.

## RESULTS

During the 10-year period, 124 cases and 372 controls matched on age and sex were identified. Forty-one percent of the participants were male and 59% were female. Males were more likely than females to use ASM (*χ^2^*[1]=13.40; *P*=0.0003).

The distribution of the type of alopecia (ie, cases) and controls by sex and ASM use is shown in Table 1. Of the cases, 110 patients had AA, 11 had AT, and 3 had AU. In the comparison of cases with any type of alopecia (AA, AT, AU) to controls (Table 2), we found no association between hair loss and ASM use (OR=0.72, 95% CI 0.41-1.26; *P=*0.2811). In the comparison of AA cases to controls, we found no significant association between ASM and AA (OR=0.66, 95% CI 0.35-1.24; *P*=0.0861). In the comparison of AT cases to controls, we found no association with ASM (OR=1.21, 95% CI 0.25-5.96; *P*=0.9999). However, when comparing AU cases to controls, we found a strong association between AU and ASM use (OR=29.52; 95% CI 1.51-577.88; *P*=0.0071).

**Table 1.** Distribution by Sex of Types of Alopecia Cases and Controls

		ADHD Stimulant Medication Use		
Patient Sex	Type of Alopecia	Yes	No	%Using ADHD Stimulant Medication
Female	Alopecia areata	6	63	8.70
	Alopecia totalis	2	2	50.00
	Alopecia universalis	0	0	0
	Control	29	190	13.24
Male	Alopecia areata	7	34	17.07
	Alopecia totalis	0	7	0
	Alopecia universalis	3	0	100.00
	Control	42	111	27.45

Note: The percentages for each row were calculated by dividing the “Yes” column of each row by the sum of the “Yes” and “No” columns for each row.

ADHD, attention-deficit/hyperactivity disorder.

**Table 2.** Relationship Between Types of Alopecia and Attention-Deficit/Hyperactivity Disorder (ADHD) Stimulant Medication Use

	ADHD Stimulant Medication Use					
Type of Alopecia	Yes	No	Total (% Yes)	Odds Ratio	95% CI	*P* Value
Any form of alopecia	18	106	124 (14.5)	0.72	0.41-1.26	0.2811
Alopecia areata	13	97	110 (11.8)	0.66	0.35-1.24	0.0861
Alopecia totalis	2	9	11 (18.2)	1.21	0.25-5.96	0.9999
Alopecia universalis	3	0	3 (100.0)	29.52	1.51-577.88	0.0071
Control	71	301	372 (19.1)	reference		

We reviewed the notes for the 3 patients with AU taking ASM. According to the patients’ medical records, no biopsies had been done to confirm the diagnosis of AU. One of the patients had a localized patch of hair loss on the right temple before progressing to AU, and another had an ophiasis pattern of AA. The third patient had pitting of several nails with no documentation of localized hair loss prior to the development of AU. The interval between starting ASM and onset of hair loss was 2 to 4 years.

## DISCUSSION

Although the number of patients in our study who experienced AU is small, and the CI for the OR between AU and ASM is large, to our knowledge, these study results are the first in the literature to suggest a potential association between AU and ASM. Additionally, we found no associations between other forms of alopecia and ASM. Aligning with published results,^13^ males in our study were more likely to be taking ASM than females, and interestingly, all 3 patients with AU were males taking ASM. In addition to diffuse hair loss, all 3 patients with AU had findings that were helpful in making the diagnosis, such as more localized patches of hair loss or nail pitting initially.

Considering the various psychiatric medications reported to be associated with AA and the results of our study, we question whether prior reports of ASM-related hair loss could also be cases of severe AA (ie, AU). We also question whether the study that found an association between ADHD and AA would have found the association restricted to AU if the various types of AA had been examined separately.^2^

Cases of hair loss related to ASM were first reported in the 1960s and were described simply as diffuse hair thinning on the vertex related to amphetamine use for weight loss.^7^ A case from 2009 describes a 5-year-old female who had been diagnosed with ADHD and took “mixed amphetamine salts” for 1 month, followed by a change of medication to lisdexamfetamine because her prior medication would “wear off” too early in the day.^11^ Five days after starting the new medication, the patient was reported to have generalized hair loss with no bald areas. Lisdexamfetamine was discontinued, and the patient's hair loss stopped 2 days later. The patient was restarted on the mixed amphetamine salts, and no further hair loss was reported. A case from 1988 describes a 57-year-old female who was treated with dextroamphetamine for narcolepsy for a period that appeared to be >10 years when she developed “rapid loss of almost all her body hair.”^12^

While the descriptions of the patients in these reports lack more specific findings of AA, the intervals between starting medication and onset of hair loss are also not classic for telogen effluvium, which is typically 3 to 4 months and is in the differential diagnosis of AU. When telogen effluvium is in the differential diagnosis but the interval does not fit, practitioners may want to consider the less classic forms of AA—AA incognita and acute diffuse and total alopecia. In general, these forms of AA have a marked female predominance and may be more common in women older than 40 years.^14^ While trichotillomania may occur in some patients with progressive AA, trichotillomania is unlikely to account for total body hair loss. Providers should also consider anagen effluvium when a patient presents with diffuse hair loss, and in the right context, recognize that the inflammation associated with AA is capable of diminishing the metabolic activity of hair follicles, thus resulting in anagen hair loss.^15^ While the classic histopathologic finding of peribulbar inflammation is helpful if present in the acute active phase of AA, biopsy findings may be less specific and less helpful as the disease progresses.^1^

The pathogenesis of AA is not completely understood and may vary depending on the trigger. Whether psychiatric conditions or the medications used to treat these conditions are associated with AA is unclear, as studies have reported conflicting results.^2-6^ Regarding medication-induced cases of AA, the culprit medication in one patient may be the cure in another. For example, while TNF-alpha inhibitors have been used successfully to treat AU, some patients have had worsening AA when placed on one of these medications.^16^ Of note, autoimmune conditions have been associated with the use of ASM, such as autoimmune hepatitis and the use of methylphenidate.^17^

ASM-related hair loss is often rapid and diffuse and is poorly understood. Our study adds to the case reports of ASM-related hair loss and represents a step toward better characterizing its clinical findings and possible etiology. Given study results, practitioners may want to ask about prior localized areas of hair loss and examine for nail pitting in patients with suspected ASM-related hair loss. Additionally, practitioners may consider monitoring patients with AA closely for progression of disease if they are taking an ASM and discontinue the ASM if disease progression develops. If possible, a period during which ASM is removed could be attempted in patients with suspected ASM-related alopecia to monitor for hair growth.

Studies with large numbers of patients with AU are required before solid conclusions can be drawn concerning the relationship between AU and ASM. Limitations of our study include questions regarding the generalizability of results beyond our geographic region, as the study was restricted to one hospital system in the southern United States. Also, we acknowledge that we have identified a possible association but did not prove causal relationship between ASM and AU, especially considering the small number of cases and that the time between starting the medicine and the onset of hair loss in our patients with AU was 2 to 4 years.

## CONCLUSION

To our knowledge, our study is the first in the literature to demonstrate a possible association between AU and the use of ASM. While this association requires confirmation, based on study results, practitioners may consider close monitoring of patients with AA who are taking an ASM for the development of worsening disease and discontinuing the medication if patients experience an increase in hair loss that appears to be progressing to AU.

## Notes

*This article meets the Accreditation Council for Graduate Medical Education and the American Board of Medical Specialties Maintenance of Certification competencies for Patient Care and Medical Knowledge.*

## References

[1] Strazzulla LC, Wang EHC, Avila L, Alopecia areata: disease characteristics, clinical evaluation, and new perspectives on pathogenesis. J Am Acad Dermatol. 2018;78(1):1-12. doi: 10.1016/j.jaad.2017.04.1141 29241771

[2] Singam V, Patel KR, Lee HH, Rastogi S, Silverberg JI. Association of alopecia areata with hospitalization for mental health disorders in US adults. J Am Acad Dermatol. 2019;80(3):792-794. doi: 10.1016/j.jaad.2018.07.044 30092332

[3] Chu SY, Chen YJ, Tseng WC, Psychiatric comorbidities in patients with alopecia areata in Taiwan: a case-control study. Br J Dermatol. 2012;166(3):525-531. doi: 10.1111/j.1365-2133.2011.10714.x 22049923

[4] Parameshwar E. Hair loss associated with fluvoxamine use. Am J Psychiatry. 1996;153(4):581-582. doi: 10.1176/ajp.153.4.581 8599419

[5] Kubota T, Ishikura T, Jibiki I. Alopecia areata associated with haloperidol. Jpn J Psychiatry Neurol. 1994;48(3):579-581. doi: 10.1111/j.1440-1819.1994.tb03017.x 7891421

[6] Perini G, Zara M, Cipriani R, Imipramine in alopecia areata. A double-blind, placebo-controlled study. Psychother Psychosom. 1994;61(3-4):195-198. doi: 10.1159/000288889 8066157

[7] Eckert J, Church RE, Ebling FJ, Munro DS. Hair loss in women. Br J Dermatol. 1967;79(10):543-548. doi: 10.1111/j.1365-2133.1967.tb11410.x 6053969

[8] Golubchik P, Sever J, Weizman A, Zalsman G. Methylphenidate treatment in pediatric patients with attention-deficit/hyperactivity disorder and comorbid trichotillomania: a preliminary report. Clin Neuropharmacol. 2011;34(3):108-110. doi: 10.1097/WNF.0b013e31821f4da9 21586916

[9] Solanto MV, Arnsten AFT, Castellanos FX, eds. Stimulant Drugs and ADHD: Basic and Clinical Neuroscience. Oxford University Press; 2001:55.

[10] Rebora A. Telogen effluvium: a comprehensive review. Clin Cosmet Investig Dermatol. 2019;12:583-590. doi: 10.2147/CCID.S200471 PMC6709511 31686886

[11] Brahm NC, Hamilton DR. Alopecia following initiation of lisdexamfetamine in a pediatric patient. Prim Care Companion J Clin Psychiatry. 2009;11(6):365. doi: 10.4088/PCC.08l00753 20098534 PMC2805578

[12] Fiedler VC. Alopecia: a relationship to amphetamine use? JAMA. 1988;259(12):1876. doi: 10.1001/jama.1988.03720120072043

[13] Oehrlein EM, Burcu M, Safer DJ, Zito JM. National trends in ADHD diagnosis and treatment: comparison of youth and adult office-based visits. Psychiatr Serv. 2016;67(9):964-969. doi: 10.1176/appi.ps.201500269 27181734

[14] Trüeb RM. Telogen effluvium: is there a need for a new classification? Skin Appendage Disord. 2016;2(1-2):39-44. doi: 10.1159/000446119 27843921 PMC5096239

[15] Kanwar AJ, Narang T. Anagen effluvium. Indian J Dermatol Venereol Leprol. 2013;79(5):604-612. doi: 10.4103/0378-6323.116728 23974578

[16] Gorcey L, Gordon Spratt EA, Leger MC. Alopecia universalis successfully treated with adalimumab. JAMA Dermatol. 2014;150(12):1341-1344. doi: 10.1001/jamadermatol.2014.1544 25322338

[17] Lewis JJ, Iezzoni JC, Berg CL. Methylphenidate-induced autoimmune hepatitis. Dig Dis Sci. 2007;52(2):594-597. doi: 10.1007/s10620-006-9525-2 17219064

